# Increasing the number of ribosomal uL6 mRNA copies accelerates aging of the budding yeast

**DOI:** 10.1007/s11033-022-08187-2

**Published:** 2022-12-28

**Authors:** Mateusz Mołoń, Monika Zaciura, Dominik Wojdyła, Eliza Molestak

**Affiliations:** 1 Department of Biology, Institute of Biology and Biotechnology, University of Rzeszów, Rzeszów, Poland; 2 Department of Molecular Biology, Maria Curie-Skłodowska University, Lublin, Poland

**Keywords:** Aging, Hac1, Lifespan, Overexpression, Paralogs, uL6 protein

## Abstract

**Background:**

Aging is a biological process from which there is no escape. Diverse factors contribute to aging, most notably cell energy metabolism. Ribosome biogenesis and translation are the two main energy-consuming processes that contribute to longevity. It has repeatedly been shown that translation disorders caused by deletion of ribosomal genes delay aging. However, the effect of increasing the amount of ribosomal proteins has remained elusive.

**Methods and results:**

We determine the relative level of the uL6A and uL6B mRNA derived from the genome and the plasmid. The appearance of additional copies of plasmid-derived uL6 leads to an increase in uL6A and uL6B derived from the BY4741 genome (mainly form B). The relative amount of mRNA of plasmid form B is several times greater than the amount of mRNA in plasmid form A. The level of mRNA derived from the plasmid is increased many times compared to the mRNA of genomic origin. Additionally, the study indicates that excess of *uL6A* is a limiting or even harmful factor in the reaction to stressful conditions. Therefore, our hypothesis states that *uL6A* transcription or mRNA *uL6A* degradation in yeast cells are tightly regulated. our data clearly demonstrate that aging is accelerated when additional copies of uL6 paralogs appear.

**Conclusion:**

Overexpression of both *uL6A* or *uL6B* accelerates aging in the budding yeast. The level of *uL6A* mRNA is tightly controlled by yeast cell. The uL6a protein plays a pivotal role in the response to environmental stress, including oxidative and osmotic stress, and thus may fall into the class of moonlighting ribosomal proteins with extra-ribosomal function.

## Introduction

The synthesis of ribosomes is one of the most energy-consuming processes in the cell. Cells must confine the production of ribosomes under conditions in which the demand for protein synthesis is decreased, e.g. calorie restriction [1]. Ribosome synthesis requires coordinated activities of all three nuclear RNA polymerases. In yeast, rRNA synthesis represents approximately 60% of total transcriptional activity and the synthesis of mRNAs encoding ribosomal proteins (RP) represents approximately 60% of all polymerase II transcription initiation events [2]. Therefore, it seems that the energetic status of the cells is crucial in ensuring its proper physiological state. The rate of metabolism, including ribosome biosynthesis, is important in many physiological processes, for instance aging [3]. In general, aging is an irreversible multifactor process that leads to destructive changes at the cell, tissue, or organism level, and consequently leads to death. Numerous intervention are known to contribute to aging in yeast, including ribosome biogenesis [4–8], anti-aging natural compounds [9, 10], mitochondrial dysfunction [11] or extrachromosomal rDNA circles formation [12, 13]. The deletion of some ribosomal proteins, particularly the 60 S subunit, is critical to achieving longevity. In the budding yeast, there are two main approaches to determine aging. Replicative aging shows the number of daughter cells produced by the yeast mother cell (budding lifespan) [14] and the lifetime of single cells (total lifespan) [15]. It is a standard model for studying the aging of mitotically active cells in higher eukaryotes, including humans. In turn, the second approach, the so-called chronological aging, refers to the length of time for which a nonbudding yeast cell retains viability during stationary phase and may model the aging of postmitotic cells [16]. Despite the abundance of data on factors and molecular pathways that may regulate aging, data on the impact of increased ribosomal protein levels on aging is elusive. Therefore, the main purpose of this study was to demonstrate the effect of overexpression of *uL6* gene paralogs on replicative aging. Here, we are the first to show that an increased number of gene copies and overexpression of uL6 paralogs lead to accelerated aging in yeast in a way independent of ER stress. Furthermore, we demonstrate that uL6a protein is strongly associated with the environmental stress response, including oxidative and osmotic stress.

## Results and discussion

### Increasing the number of uL6 copies in yeast cells has physiological and molecular impact

**Fig. 1:**
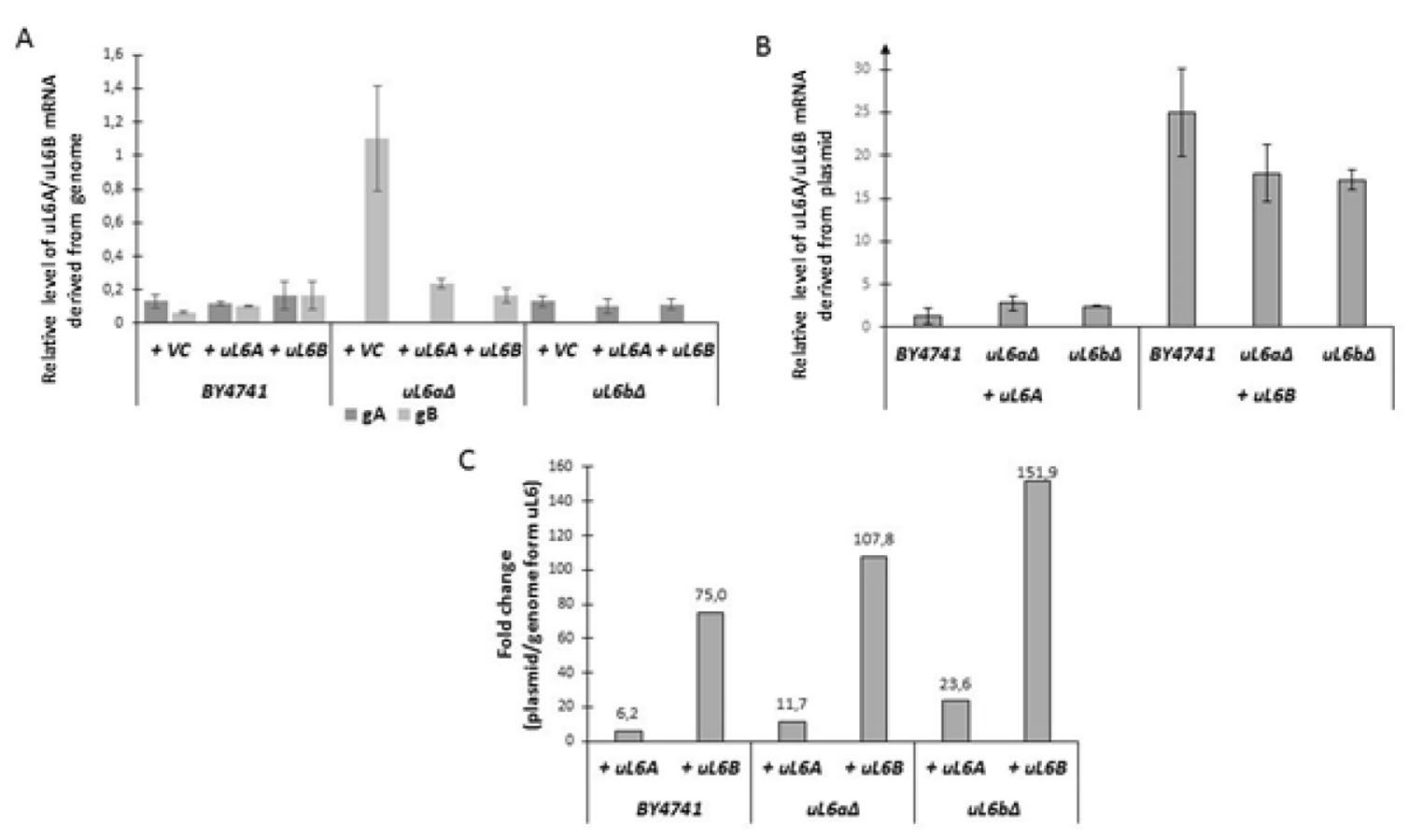
Relative level of *uL6A/uL6B* mRNA derived from genome (gA, gB) (**A**) and plasmid (+ uL6A, (+ uL6B) (**B**). Fold change of plasmid/genome form uL6 (**C**). Error bars represent standard deviations obtained from three independent experiments. VC - empty vector control

Our recent data showed that lack of the *uL6A* or *uL6B* isoform significantly depletes the translation efficiency and polysome profile, revealing the presence of the so called half-mers in both single deletion paralogs *uL6* and simultaneously extending longevity. Furthermore, we demonstrated that addition of copies of the uL6 protein restored the wild-type phenotype in all of the analyzed strains (regardless of the A or B isoform) [6]. Therefore, here we checked the relative level of the *uL6A* and *uL6B* mRNA derived from the genome and the plasmid. As shown in Fig. 1 A, the mRNA levels of the genomic-derived paralogs are similar in the wild-type strain BY4741. The level of form B is slightly lower than that of form A under control conditions (empty vector), but it increases with overexpression of form A or B. Interestingly, the appearance of additional copies of plasmid-derived uL6 leads to an increase in *uL6A* and *uL6B* derived from the BY4741 genome (mainly form B). The level of the *uL6B* genomic form is increased several times in the *uL6aΔ* mutant under control conditions compared to the wildtype strain. As a result of the presence of additional copies of the A or B *uL6* genes in the mutant, the level of *uL6B* mRNA is reduced by approximately 80% compared to the control (*uL6aΔ* + VC). The level of genomic form A mRNA in the *uL6bΔ* mutant is constant (irrespective of appearance of additional *uL6A* or *uL6B* gene) and comparable to BY4741 (Fig. 1 A).

**Fig. 2:**
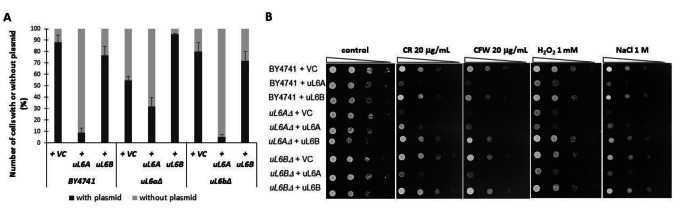
Frequency of plasmid pCM190 (VC), + uL6A, + uL6B loss in BY4741, *uL6AΔ* and *uL6BΔ* strains after 3 passages on rich YPD medium (without selection). Results were obtained by analyzing 300 independent clones in three biological repeats (**A**). Phenotype screening analysis of yeast mutant strains exposed to different environmental conditions. Yeast was grown in SD-ura medium, spotted onto SD-ura plates containing the indicated amounts of Congo red (CR), calcofluor white (CW), hydrogen peroxide (H_2_O_2_), sodium chloride (NaCl), and incubated at 28 °C. Growth on SD-ura agar plates was treated as a control. Representative results from three independent experiments are shown (**B**). Error bars represent standard deviations obtained from three independent experiments

Next, we determined the mRNA levels of both paralogs derived from plasmids. This seems to be pivotal in the context of tightly controlling ribosome synthesis, because as shown previously, the appearance of an additional form of *uL6A* or *uL6B* reverts the mutant phenotype back to the wild-type [6]. As shown in Fig. 1B, the level of plasmid form A in wild-type and both deletants is similar. Surprisingly, the relative amount of mRNA of plasmid form B is several times greater than the amount of mRNA in plasmid form A, e.g. 18.3 times in the wild-type and about 6.5 time in mutants. We then estimated the fold change of the plasmid/genomic form *uL6*. As seen in Fig. 1 C, the level of mRNA derived from the plasmid is increased many times compared to the mRNA of genomic origin. We noticed the greatest changes during overexpression of the *uL6B* form. In the case of the *uL6bΔ* mutant complemented by the plasmid carrying the *uL6B* gene, the mRNA level increase was almost 152-fold, while in the *uL6aΔ* strain the increase was almost 108-fold and in the wild-type strain at least 75-fold. In turn, among the fold changes in the *uL6A* form, the mRNA level increased 6 times in BY4741, 12 times in *uL6aΔ*, and almost 24 times in *uL6bΔ*. The inevitable consequence of the increase in the number of copies of the *uL6* genes is the increase in the level of mRNA in cells. It is surprising that despite the use of an identical plasmid (pCM190), the transcription level of *uL6B* is significantly higher than that of *uL6A*. Therefore, we used the plasmid loss and toxicity tests to demonstrate that a significantly lower level of form A uL6 is associated with protein toxicity. As shown in Fig. 2 A, the presence of two versions of *uL6A* (i.e. genomic and plasmid simultaneously – wild-type and *uL6bΔ*) leads to loss of plasmid shortly after growing in a rich medium (three passages). For BY4741 and *uL6bΔ*, a mere less than 10% of the cells had a plasmid after passaging and were then able to grown in the SD-ura medium. In the case of overexpression of *uL6B*, we either did not notice any significant changes or the effect was positive (e.g. *uL6aΔ*) compared to the control. Additionally, as shown in Fig. 2B, increase in *uL6A* mRNA levels leads to increase in the sensitivity to osmotic stress (NaCl), oxidative stress (hydrogen peroxide) and the level of cell wall biosynthesis inhibitors (Congo red and Calcofluor white). Thus, this study suggests that *uL6*, especially *uL6A*, plays a pivotal role in cell wall biosynthesis or remodeling as well as in response to environmental stress. Additionally, the study indicates that excess of *uL6A* is a limiting or even harmful factor in the reaction to stressful conditions. Therefore, our hypothesis states that *uL6A* transcription or mRNA *uL6A* degradation in yeast cells are tightly regulated.

**Fig. 3:**
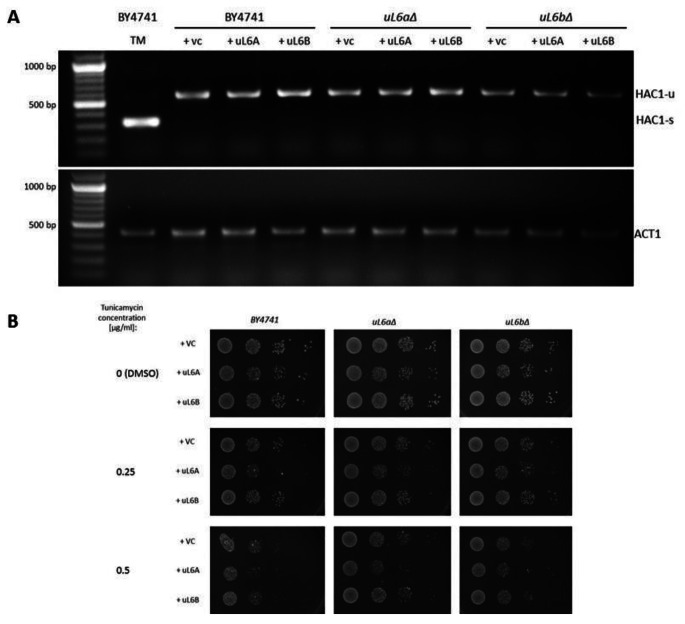
Analysis of *HAC1* mRNA splicing in yeast strains (**A**). In each sample, *ACT1* mRNA was analyzed as a control. WT (BY4741) treated with tunicamycin (TM) for 1 h at the concentration of 5 µg/mL was used as a positive control of spliced *HAC1* mRNA represented by the 325 bp band (HAC1-s). Unspliced *HAC1* mRNA (HAC1-u) is represented by the 577 bp long PCR product (**A**). Sensitivity to tunicamycin of strains BY4741, *uL6aΔ*, *uL6bΔ* with pCM190 (VC), +ul6A and + ul6B plasmids assayed by spot test (**B**)

In general, physical stress such as increased translation load can lead to an imbalance between the demand for protein folding and the capacity of the ER to fold proteins, resulting in ER stress. ER stress occurs when the capacity of the ER to fold proteins becomes saturated [17]. We then posed the question of whether excess of ribosomal uL6 proteins leads to endoplasmic reticulum (ER) stress. For this purpose, we used two approaches: the analysis of *HAC1* mRNA splicing and spot test against cells treated with tunicamycin (a nucleoside antibiotic used as the ER stress inducer). The data in Fig. 3 A clearly demonstrate that increasing *uL6* paralog levels do not cause ER stress. In the case of all analyzed strains, a PCR product 577 bp long was obtained, which is a marker for the unspliced *HAC1* mRNA. In turn, the 325 bp long PCR product for spliced mRNA was obtained only in the positive control. We confirmed the splicing test results by means of a spot test as shown in Fig. 3B. Therefore, we conclude that increasing the amount of mRNA, regardless of the form of uL6, leads to stress in a manner independent of Hac1. These are surprising results that require further thorough analysis in future, especially with regard to regulation of the *uL6A* mRNA level in cells.

### Additional copies of uL6A or uL6B genes accelerate aging

**Fig. 4:**
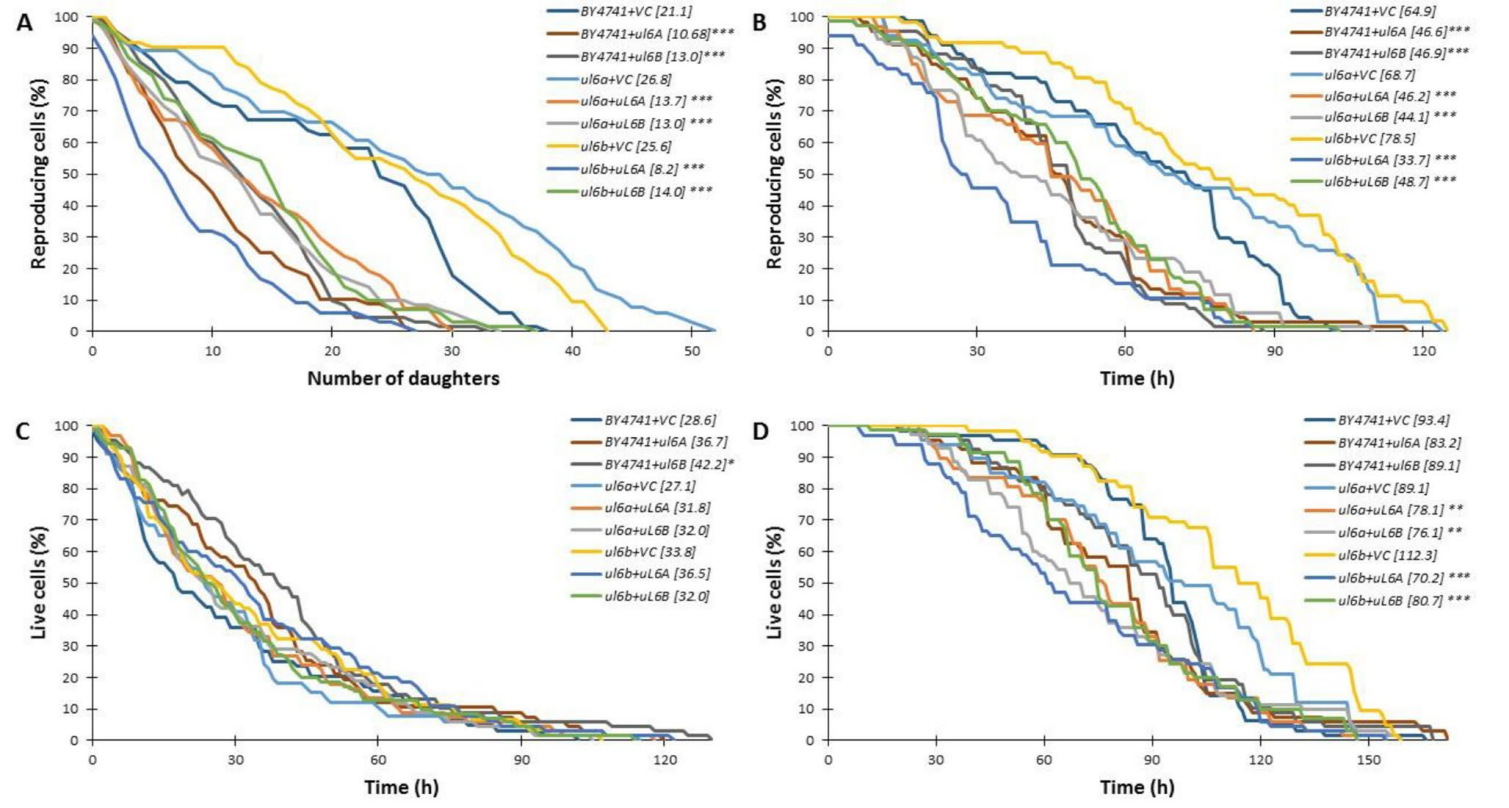
Comparison of the budding lifespan (**A**), reproductive lifespan (**B**), post-reproductive lifespan (**C**) and total lifespan (**D**) of the wild type and single *uL6aΔ* or *uL6bΔ* deletion mutants with overexpression of uL6A or uL6B after cultivation on solid SD-ura media. Statistical significances were assessed using ANOVA and Dunnett’s post hoc test (**p < 0.05, ***p < 0.001). Data represent mean values from two independent experiments. VC indicates empty vector control

Aging is an inner biological process from which there is no escape [18]. Understanding cellular aging occurs may also help modulate human senescence. Therefore, the main aim of the current research in biogerontology is to answer the question of how to live longer with a good healthspan. Numerous studies of model organisms have shown surprising similarities in fundamental biology of cells and mechanisms of human aging. In this sense, research into lower organisms, including unicellular yeast, remains relevant for the understanding of human aging. Yeast is useful in identifying the evolutionary conserved aging pathways that are common in various organisms, among others the worm, fruit fly, mouse, and human. In addition, easy genetic manipulations, fast growth and short cell cycle make yeast a workhorse model organism to study longevity. Overall cell energetics and the molecular pathways involved in the high energy-consuming ribosome biosynthesis process are closely related to aging. Previous data demonstrated a correlation between the decreased efficiency of the translational apparatus and the longevity of yeast suggesting the cell metabolism rate as a possible factor regulating the lifespan [3]. It seems that maintaining the steady state of gene expression is crucial to maintain the energy balance of the cell. It has been demonstrated recently that limiting the levels of mRNA essential for the replication genes (*ORC1-6*) in a heterozygous system leads to a significant delay in aging [19]. Our previous data show that *uL6* is not essential for yeast cell survival, although lack of this protein depletes the growth rate and disturbs budding. We also suggested that the two *uL6* isoforms most likely serve the same purpose, playing a pivotal role in the adaptation of the translational machinery performance to the metabolic activity of the cell. The deletion of a single *uL6* gene significantly prolongs the lifespan, but only in cells grown on rich medium with a high metabolic rate [6]. Because the analysis of aging parameters showed a significant effect of the uL6 deletant, we asked whether overexpression of *uL6A* and *uL6B* genes in the wild-type strain and single deletion mutants would change the budding lifespan or the total lifespan. For the construction of the strains, the standard expression vector pCM190 [20] with the uracil marker was used. The lifespan analysis was performed using the minimal medium (SD-ura). In this experimental setup, the controls are wild-type, *ul6aΔ* or *ul6bΔ* mutants. As can be seen in Fig. 4 A, overexpression of both the *uL6A* and *uL6B* genes leads to a statistically significant decrease of the budding lifespan for all analyzed strains (*p* < 0.001) compared to control. Surprisingly, complementation of the *uL6A* or *ul6B* genes in mutants did not cause returning to the wild-type budding lifespan, as expected on the basis of the data from the ribosome profile. A consequence of the budding lifespan reduction is also a significant decrease in reproductive lifespan (*p* < 0.001) (Fig. 4B).

Interestingly, the post-reproduction lifespan (time between the last budding and cell death) was significantly changed (increased) only in the case of the *ul6B* gene overexpression in the wildtype BY4741 strain (*p* < 0.05) (Fig. 4 C). Then, we determined the total lifespan of the cells (time between the cell birth and death). Our analyses show that an additional copy of the gene or complementation of the *ul6aΔ* or *ul6bΔ* mutants can lead to a significant reduction in the total lifespan, namely *p* < 0.01 and *p* < 0.001, respectively. In the case of BY4741, the total lifespan was also decreased during overexpression of uL6A or uL6B paralogs, but without statistical significance (Fig. 4D). Thus, our data clearly demonstrate that aging is accelerated when additional copies of uL6 paralogs appear. Importantly, a significant acceleration of aging was also observed during chronological aging analysis, which suggests that *uL6* overexpression may be a key factor limiting the longevity of both mitotically active and post-mitotic cells (data not shown). Here we also confirmed that single mutants containing the control vector had a significantly increased budding lifespan compared to wild-type, showing that the glucose source of carbon is more likely to be a critical factor in longevity rather than growing in rich medium (cells were grown in poor medium). Earlier studies on the paralog pair did not show conclusively that divergent budding lifespan phenotypes among paralog pair deletion strains may be explained by the fact that one paralog is transcribed at a higher level than the other [21]. Thus, uL6 proteins have been implicated in numerous biological events, beyond translation, with extra-ribosomal function or moonlighting activity accentuating their functional heterogeneity.

## Materials and methods

### Strains and growth conditions

All yeast strains used in this study are listed in Table 1. The single *ul6aΔ* or *ul6bΔ* mutant and wild-type BY4741 strains were obtained from the EUROSCARF (Oberursel, Germany).

Plasmids for the complementation of *uL6A* and *uL6B* were generated on a basis of a tetracycline repressive pCM190 vector [20], using standard genetic techniques. Yeast cells were grown in SD medium containing 2% (w/v) glucose (POCH), supplemented with L-histidine (20 mg/L) (Sigma-Aldrich), L-leucine (60 mg/L) (Sigma-Aldrich) and L-methionine (20 mg/L) (Sigma-Aldrich) on a rotary shaker at 150 rpm or on solid rich SD medium containing 2% agar. The experiments were performed at 28 °C.

**Table 1:** Strains used in this study

Strain	Genotype	Source
*BY4741 + VC*	MATa; *his3Δ1; leu2Δ0; met15Δ0; ura3Δ0* [pCM190]	[6]
*BY4741 + uL6A*	MATa; *his3Δ1; leu2Δ0; met15Δ0; ura3Δ0* [pCM190-uL6A]	[6]
*BY4741 + uL6B*	MATa; *his3Δ1; leu2Δ0; met15Δ0; ura3Δ0* [pCM190-uL6B]	[6]
*ul6aΔ + VC*	MATa; *his3Δ1; leu2Δ0; met15Δ0; ura3Δ0; YGL147c::kanMX4* [pCM190]	[6]
*ul6aΔ + uL6A*	MATa; *his3Δ1; leu2Δ0; met15Δ0; ura3Δ0; YGL147c::kanMX4* [pCM190-uL6A]	[6]
*uL6aΔ + uL6B*	MATa; *his3Δ1; leu2Δ0; met15Δ0; ura3Δ0; YGL147c::kanMX4* [pCM190-uL6B]	[6]
*uL6bΔ + VC*	MATa; *his3Δ1; leu2Δ0; met15Δ0; ura3Δ0; YNL067w::kanMX4* [pCM190]	[6]
*uL6bΔ + uL6A*	MATa; *his3Δ1; leu2Δ0; met15Δ0; ura3Δ0; YNL067w::kanMX4* [pCM190-uL6A]	[6]
*uL6bΔ + uL6B*	MATa; *his3Δ1; leu2Δ0; met15Δ0; ura3Δ0; YNL067w::kanMX4* [pCM190-uL6B]	[6]

### Determination of budding lifespan

After overnight growth, the cells were arrayed on a YPD plate using a micromanipulator. The budding lifespan was determined microscopically by a routine procedure with the use of a micromanipulator as described previously [22]. The number of buds formed by each mother cell reflects its reproductive potential (budding lifespan). In each experiment, 45 single cells were analyzed. The results represent measurements for at least 90 cells analyzed in two independent experiments. The analysis was performed by micromanipulation using the Nikon Eclipse E200 optical microscope with an attached micromanipulator.

### Determination of the total lifespan

The total lifespan is the length of life of a single mother yeast cell expressed in units of time and was calculated as the sum of reproductive (time between the first and the last budding) and post-reproductive lifespans (time from the last budding until cell death). The total lifespan of the *S. cerevisiae* yeast was determined as previously described by [15] with small modification [22]. Ten-microliter aliquots of fresh exponential culture of yeast (OD_600_ = 0.5–0.7) were collected and transferred on YPD (**y**east extract (Difco) **p**eptone (Difco) **d**extrose (POCH)) plates with solid medium containing Phloxine B (10 µg/mL). In each experiment, 45 single cells were analyzed. During manipulation, the plates were kept at 28 °C for 15 h and at 4 °C during the night. The results represent measurements for at least 90 cells analyzed in at least two independent experiments. The analysis was performed by micromanipulation using the Nikon Eclipse E200 optical microscope with an attached micromanipulator.

### Quantification of the level of genomic and plasmid uL6 mRNA A and B isoforms

Yeast cells were grown to the logarithmic phase (OD_600_ = 0.5–0.7) in SD medium with glucose and amino acids without uracil and then harvested by centrifugation at 3000xg and flash-frozen in liquid nitrogen. Total RNA was extracted from the cells following a previously developed method [23]. The RNA concentration was measured with a NanoDrop2000c spectrophotometer (Thermo Scientific). The RNA samples obtained were subjected to DNase treatment with a TURBO DNA-free kit according to the manufacturer’s protocol (AM1907, Ambion). 500 ng of RNA were used as a template for cDNA synthesis reaction which was set up in the volume of 20 µl using 200 U of SuperScript IV Reverse Transcriptase (18,090,050, Invitrogen). 10x Reverse Transcripton Random Primers (Applied Biosystems) were used in the reaction.

For quantification of genomic *uL6A* isoform mRNA, g_uL6A_F and g_uL6_univ_R primers were used (amplification efficiency – 90.5%), for B isoform - g_uL6B_F and g_uL6_univ_R (amplification efficiency – 89.5%). Plasmid-derived uL6 mRNA level was quantified with the use of pl_uL6A_F and pl_uL6_univ_R primers for A isoform (amplification efficiency – 86%) and pl_uL6B_F and pl_uL6_univ_R primers for B isoform (amplification efficiency – 76%). *ACT1* gene was used as the reference gene and was amplified with the use of ACT1_F and ACT1_R primers (amplification efficiency – 95.5%). All primers are presented in the Table 2. Quantitative PCR was performed using the StepOnePlus Real-Time PCR System (Applied Biosystems) and Power SYBR Green PCR Master Mix (4,368,577, Applied Biosystems). 10-µl reactions were set up using 2.5 µl of template from the cDNA synthesis reaction, diluted 16-fold from the Real-Time (RT) reaction and primers with a final concentration of 200 nM. Reaction mixtures were set up in three repetitions on 96-well plate (4,346,906, Applied Biosciences) together with “no template control” (NT) and “no reverse transcription” (noRT) control. Plates with reaction mixtures were sealed with adhesive cover (4,360,954, Applied Biosystems), centrifuged and subjected to qPCR reaction. The thermal cycling conditions included an initial denaturation step at 95 °C for 10 min, followed by 40 cycles at 95 °C for 15 s and 60 °C for 1 min. The specificity of reaction was confirmed by melting curves of PCR products. Data quantification was performed on the basis of the mathematical model presented by [24] with further modifications [25] to provide efficiency of amplification - E. For all samples the value of E_ref_^Cqref^ (obtained based on reference PCR product) was divided by E_target_^Cqtarget^ (obtained based on target PCR product) and the ratio was regarded as the relative level of certain *uL6* mRNA form. All data were analyzed with StepOne Software v2.2 (Applied Biosystems).

**Table 2:** Sequences of primers used for quantification of uL6 mRNA.

Primer name	Template	Sequence (5’- 3’)
g_uL6A_F	Genome *uL6*	GAAGCCCGTACCAGAAGTTC
g_uL6B_F		CAAACTCTAGCCTCCAATAGTCAC
g_uL6_univ_R		ACAACCTTGACGATTCTGGAC
pl_uL6A_F	Plasmid *uL6*	GTTTCTCACAAGGGTTTTATTACTGAAGATTTA
pl_uL6B_F		AGGGTTTCATTGTCGAAGACATG
pl_uL6_univ_R		GGGCGTGAATGTAAGCGTGA
ACT1_F	Genome *ACT1*	TCACGCCATTTTGAGAATCG
ACT1_R		TTCAGCAGTGGTGGAGAAAGAG

### Analysis of HAC1 mRNA splicing

cDNA was synthesized as described in the previous paragraph. The analysis of the *HAC1* splicing was conducted as previously described [26]. cDNA was used as a template for PCR reaction and products were analyzed on 1% agarose gel. Primers used for the analysis are listed in Table 3. The amplification with the use of primers HAC1_F and HAC1_R results in products 577 bp long for unspliced Hac1 mRNA and 325 bp long for spliced mRNA. Primers ACT1_contr_F and ACT1_contr_R allow for amplification of DNA fragment 431 bp long. The temperature of annealing step was determined for 60ºC.

**Table 3:** Sequences of primers used for analysis of *HAC1* mRNA splicing

Primer name	Template	Sequence (5’- 3’)
HAC1_F	*HAC1*	CACTCGTCGTCTGATACG
HAC1_R		CATTCAATTCAAATGAATTCAAACCTG
ACT1_contr_F	*ACT1*	CTGGTATGTTCTAGCGCTTG
ACT1_contr_R		GATACCTTGGTGTCTTGGTC

### Plasmid loss assay

Control strains (BY4741, *uL6aΔ*, *uL6bΔ*) was first transformed (high-efficiency *S. cerevisiae* lithium acetate transformation) with the appropriate plasmids (pCM190 + VC; pCM190 + uL6A; pCM190 + uL6B). The resultant strains were passaged three times on rich YPD medium (without selection). The cells were then plated and the resulting colonies were transferred to liquid selection medium (SD-ura) in multi-well plates. Plates were grown with shaking for 48 h at 28 ^o^C. The number of clones growing in SD-ura medium was counted, and the frequency of plasmid loss was determined.

### Phenotypic analysis—a spot test for sensitivity to Congo Red, Calcofluor White, sodium chloride and hydrogen peroxide

The yeast cultures were grown to the exponential phase in SD-ura medium (OD_600nm_ between 0.5 and 0.7) and serially diluted to different cellular concentrations as indicated (dilution ratio: 1:10, 1:100, 1:1000, 1:10000). Five microliters of each cell suspension were spotted onto agar plates containing various concentrations of Congo red (2 µg/mL) (Sigma-Aldrich), Calcofluor White (20 µg/mL) (Sigma-Aldrich), sodium chloride (1 M) (Sigma-Aldrich), hydrogen peroxide (1 mΜ) (POCH) and Tunicamycin (0.25 and 0.5 µg/mL) (Sigma-Aldrich). Growth was registered 48 h after incubation at 30 ^o^C. All phenotypes described in this work were confirmed by at least three independent experiments.

### Statistical analysis

The results represent the mean ± SD values for all cells tested in two independent experiments. The differences between the wild-type and the isogenic mutant strains were estimated using one-way ANOVA and Dunnett’s post-hoc tests. The values were considered significant when * *p* < 0.05; ** *p* < 0.01, *** *p* < 0.001. The statistical analysis was performed using the Statistica 10 software (StatSoft Inc., Tulsa, OK, USA).
